# Determining an Appropriate Outcome Measure in Neurosurgical Research: Investigating Meaningful, Valid, and Practical Metrics

**DOI:** 10.7759/cureus.5610

**Published:** 2019-09-09

**Authors:** Christopher Louie, Erin N D'Agostino, David Han, Timothy C Ryken

**Affiliations:** 1 Neurosurgery, Mayo Clinic, Jacksonville, USA; 2 Neurosurgery, Geisel School of Medicine at Dartmouth, Lebanon, USA; 3 Urology, Geisel School of Medicine at Dartmouth, Lebanon, USA; 4 Neurosurgery, Dartmouth Hitchcock Medical Center, Lebanon, USA

**Keywords:** outcomes, measures, validity, neurosurgical study methodology

## Abstract

Given the rapidly evolving pace of research and technology in the neurosurgical field, it is critical to consider the parameters of valid, practical, and meaningful study outcome measures. Here we review fundamental aspects of selecting outcome measures in the context of neurosurgical research. Exemplifying work in meningiomas and high-grade gliomas, we delineate a proposed framework for identifying an appropriate outcome measure. Four fundamental components of an outcome measure are defined and characterized: understanding characteristics of a good outcome measure; developing a research question to address an outcome measure; defining the outcome measure, and considering limitations of an outcome measure. This four-part framework enhances and promotes the methodology for determining if an outcome measure is valid, practical, and ultimately meaningful.

## Introduction and background

Evaluating the appropriateness and utility of an outcome measure is paramount to formulating and critiquing study conclusions. However, it is challenging to determine whether an outcome measure appropriately follows and elucidates the central question of a research study.

The field of neurosurgery, in its nascency relative to other surgical disciplines, is rapidly evolving through novel treatment approaches, technological innovations, and enhanced understanding of neurologic diseases. Identifying a meaningful outcome measure is potentially daunting in a field laden with complex (and often heretofore unknown) disease pathology, multidisciplinary treatments, and elusive stages of disease progression. Efforts are underway to standardize research methodologies, demonstrated by the ongoing development of the Core Outcome Measures and Effectiveness Trials (COMET), an initiative created for fostering comparability and methodological appropriateness of research outcomes [1].

**Figure 1:**
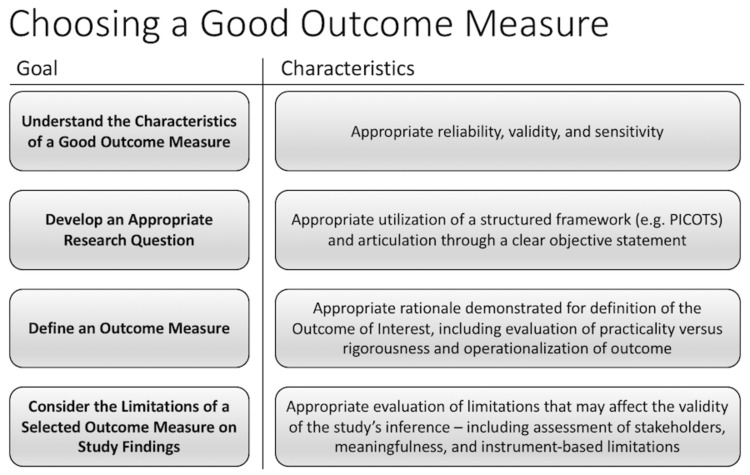
Conceptual Framework for Determining an Outcome Measure PICOTS: Population/Problem, Intervention/Exposure, Comparison, Outcome, Timeframe, and Setting.

In an effort to similarly contribute to the discussion on research outcomes methodology in neurosurgical research, we provide a basic framework for identifying an appropriate and meaningful outcome measure. Using research in meningioma and high-grade glioma as examples, we delineate a process for identifying an appropriate outcome measure into four components: understanding characteristics of a good outcome measure, developing a research question to address an outcome measure, defining an outcome measure, and considering limitations of a selected outcome measure (Figure 1). This four-part framework can help determine whether a research outcome measure is valid, practical, and ultimately meaningful.

## Review

Understanding the characteristics of a good outcome measure

An outcome measure is broadly defined as the measurable unit of analysis to evaluate clinically important changes in a study sample after an exposure or intervention over time [2]. Disease processes like meningioma and high-grade glioma, while both primary brain tumors, may be best evaluated by outcome measures that are substantially different. Examples range from objective endpoints like one-year mortality, or more subjective measures like self-reported quality of life. Regardless of whether the outcome measure is objective or subjective, there are inherent characteristics to consider when selecting an outcome measure, namely an outcome’s reliability, validity, and sensitivity.

Reliability (generally synonymous with reproducibility and repeatability) describes the extent an instrument obtains the same results when repeated, keeping the measurement conditions the same [3]. Reliability also depicts how well the tool will demonstrate a different result when measurement conditions differ. Types of reliability include inter-rater reliability and test-retest (intra-rater) reliability. In the context of survey research, inter-rater reliability indicates concordance across two separate raters answering the same question [3]. An example includes two neurosurgeons reading the same series of MRIs and how well they agree on the findings independently; more consistent results from both raters would indicate greater inter-rater reliability. Test-retest reliability (or intra-rater reliability) refers to the consistency in responses a participant provides in the same survey taken at two distinct occasions (i.e., at the study outset and several weeks later) [4]. Test-retest reliability can be problematic in the brain tumor population given that self-reported items may be affected by the patient’s ability to consistently complete items. This is the case with health-related quality of life measured by the Functional Assessment of Cancer Therapy-brain (FACT-Br) and the European Organization for Research and Treatment of Cancer (EORTC) Brain Cancer Module QLQ-BN20 [5]. These two measures are specifically designed for patients with brain tumors, but both have demonstrated inconsistent reliability, likely secondary to limitations in patient ability to self-report consistently [6-8].

Validity characterizes the extent an outcome measure captures what it is intended to measure. Reliability is related to validity in that reliability is a necessary but not sufficient component of validity. There are various types of validity, including face validity, construct validity, content validity, and criterion validity. Face validity is the most subjective measure of validity and refers to the general sentiment of whether or not the questionnaire seemingly measures what it is intended to, as perceived by study participants [3]. Construct validity characterizes the appropriateness of inferences made from the instrument measures on its intended subject [4]. If an expert in the field deems that an instrument (i.e., questionnaire) indeed measures the topic of interest (supported by standardized statistical models or methodology), then that type of validity is termed content validity. Criterion validity is the most objective measure of validity and characterizes how well a test measure correlates with the outcome of another test measure [9]. The Karnofsky Performance Score, discussed further below, is a well-established scale that is often used to validate tools characterizing functional status in cancer patients, such as elucidating ability to complete daily tasks, clinical changes, and quality of life [10]. Additionally, Evans’ index is an accepted proxy for planimetric measurement of cross-sectional ventricular and cortical areas, and has been used to estimate ventricle size since 1942 [11-12]. Therefore, Evans’ index attains face validity (generally perceived as a valid proxy) and criterion validity (accurate use as a proxy measure). However, there is often no gold standard or statistical models validating such measurements, so content validity would not be attainable. Investigators establish construct validity by confirming that an instrument behaves as expected with their data, supporting their conclusions and assumptions [9]. For example, an investigator may hypothesize that a patient with end-stage glioblastoma multiforme (GBM) would score lower on the mini-mental status exam (MMSE) given presumed cognitive decline-one of the most commonly utilized neurocognitive tests in brain cancer research. Supporting this hypothesis with data would help establish construct validity.

Finally, sensitivity and responsiveness generally describe the extent to which an instrument can detect change. Sensitivity refers to the capacity of an instrument to measure objective changes, where responsiveness refers to the capacity to detect clinically relevant change [13]. Regarding sensitivity, while the MMSE has been noted to predict tumor progression, it has also been characterized as insensitive to other significant events in brain cancer, such as cognitive slowing [8,14]. This is exemplified by the finding that decreased global neurocognitive performance while taking anti-epileptic drugs is not necessarily associated with decreasing scores on MMSE [15].

Developing a research question

Concomitant with selecting an outcome measure, it is important to articulate an appropriate research question that addresses and elucidates the variable of interest. One conventional tool in evidenced-based medicine for systematic reviews and meta-analyses is the PICO question framework: Population/Problem, Intervention/Exposure, Comparison, and Outcome [16]. A well-known extension of this has been created by including Timeframe and Setting (PICOTS). While not all components of the template may be relevant to every question (i.e., observational studies without a comparator arm), the basic PICOTS method allows investigators to hone a research question or objective of interest.

Applying the framework to meningioma, an investigator may inquire: “How do survival rates (Outcome) compare with and without resection (Intervention and Comparison) in adults with asymptomatic meningioma (Population) at five years (Timeframe) in the United States (Setting)?” For high-grade glioma, a research question could be: “How does health-related quality of life (Outcome) compare with and without resection (Intervention and Comparison) in patients with recurrent GBM (Population) at one year (Timeframe) in urban American cities (Setting)?”

Defining the outcome measure

Once an outcome of interest is articulated and a research question is established, an outcome must by operationalized into a discrete unit-the outcome measure. Factors that must be considered in operationalizing an outcome measure include: adequate time of the study period to capture appropriate outcomes (i.e., scope), acceptable sample size for statistical significance (i.e., power), sufficient resources to carry out the study with respect to scope and power (i.e., practicality), and the potential benefit for patients (i.e., relevance) [17].

In the above example of meningioma, the outcome of interest was “survival rates.” Survival rate is a common outcome measure favored for its objectivity. Furthermore, the parameters of this seemingly standard outcome require defining. If an investigator is conducting a randomized controlled trial, investigating one-year survival with a particular treatment, this could be defined as the proportion of patients in the cohort alive one year after beginning the observation period. However, it is necessary to consider whether a one-year survival follow-up period is adequate to observe effects in the study arms for this disease that generally has a positive prognosis. For patients with a slow growing tumor (i.e., meningioma) a year is likely not long enough to elucidate appreciable differences between study arms. It may therefore be worth considering five-year or ten-year follow-up periods, and facing the correlating trade-off of additional cost and resources. The sample size required to generate an adequate outcome of interest may also limit feasibility, given the need for statistical significance (i.e., power). Therefore, the practicality of an outcome measure (i.e., relevance to patients) must ultimately be considered, as a ten-year follow-up time may be appropriate, yet it may require too much time and resources to complete, too many patients may be lost to follow-up, and it may not yield a significant benefit for patients. Clearly the value of an outcome measure-characterized by the study scope, power, practicality, and relevance-vary widely for a given disease and patient population.

The considerations of “practicality” further extends to subjective patient-reported outcome measures like “quality of life,” such as in the example study of GBM. The FACT-Br has been demonstrated as a valid measure of health-related quality of life for patients with high-grade glioma after resection, but at least one study reported difficulty utilizing the instrument secondary to the 46-item questionnaire length [7,18]. Some survey tools are utilized specifically for their ease of administration, like the MMSE. However, as mentioned, the MMSE may be insensitive to changes in global neurocognitive performance in patients taking anti-epileptic drugs. Similarly, the “three question depression” score is sometimes used as a brief but somewhat insensitive measure for depression in brain cancer [19]. Therefore, the appropriate application of clinical tools is a significant factor when considering practicality and necessary resources.

Whether measuring objective outcomes (i.e., one-year survival) or subjective outcomes (i.e., self-reported quality of life), equally important to how the outcome of interest is defined is the methodology for operationalizing the outcome measure: namely, the correlation between the outcome of interest and the specified outcome measure. For example, if the outcome of interest is a high level of functional independence, an outcome measure of survival is not clearly apropos (the Functional Independence Measure would be more suitable) [20]. Additionally, some studies have used the European Organization for Research and Treatment of Cancer (EORTC) QLQ C-30 to evaluate quality of life in patients with brain tumors; however, this tool is designed for a more general quality of life assessment for cancer patients and has minimal utility for identifying issues specific for patients with brain tumors (i.e., presence of seizures) [21]. Therefore this tool would have limited capacity for characterizing quality of life for individuals with brain tumors. Instruments that are broad, like the “three question depression” scale, MMSE, and EORTC QLQ C-30, while frequently used and validated in general populations, likely do not demonstrate fidelity to the brain tumor population as well as instruments that are more disease-specific, such as the FACT-Br or EORTC QLQ BN20. However, as mentioned, there may be a trade-off between the practicality (i.e., funding, ease of administration, time etc.) and the ideal outcome measure that maximizes reliability, validity, and sensitivity. Regardless of the rationale, the outcome of interest and methodology for operationalizing the outcome measure requires transparency.

Considering the limitations of the selected outcome measure

Limitations of outcome measures include the difference between clinically meaningful and statistically significant effect sizes, the appropriateness of the outcome measure to the study population, the generalizability of findings to relevant patient cohorts, and the limitations inherent in the instrument(s) used to gather data. Ideally, study findings are both clinically meaningful and statistically significant. If a finding is clinically meaningful with a large effect size across cohorts but is not statistically significant, then the study may be underpowered, and a larger study may be warranted. A statistically significant finding with a modest effect size may not be clinically meaningful (or may demonstrate equipoise across the study cohorts), which should be considered when deducing clinical relevance. Across many studies, particularly those focused on patient outcomes from spine procedures, there is increased emphasis on the “minimal clinically important difference” (MCID) [22]. This value serves as a critical threshold for measuring treatment effectiveness (most commonly, meaningful improvement reported by the patient), used pragmatically to determine whether a procedure is deemed beneficial. The Karnofsky Performance Status Scale (also known as the Karnofsky Performance Score, or “KPS”) is an often implemented tool to characterize functional status and ability to independently complete activities of daily living among cancer patients; KPS score is also often used to inform treatment regimens, measure changes in patient functionality, and is often a proxy for MCID and quality of life in oncological studies [23].

Additionally, the definition of a “meaningful and relevant” outcome differs based on the stakeholders (i.e., patients, providers, researchers, insurance companies, hospital administrators, etc.). When considering outcome measure limitations, identifying the perspective of these various population interests may be productive-especially with regard to what is meaningful to the patient, vis-a-vis the MCID. Funding by agencies like the Patient-Centered Outcomes Research Institute (PCORI) promote partnerships between researchers and patients to find measures that meet the needs of multiple involved stakeholders.

The two exemplified disease processes-meningioma and high-grade glioma-illustrate a significant difference in meaningful outcome. In the context of high-grade glioma, where prognosis is poor and success is measured by prolonged life on the order of months, quality of remaining life becomes increasingly significant to patients. Treatment of high-grade glioma may modestly prolong survival but may also cause severe side effects that decrease quality of life. Therefore, depending on the patient’s goals of care, survival may not be the most meaningful outcome.

The limitations of the historical endpoints of tumor research, namely overall and progression-free survival, were acknowledged and discussed at the 2006 FDA-AACR (American Association for Cancer Research) Brain Tumor Endpoint Workshop. The workshop reported a need for outcome measures that are more representative of quality of remaining survival. Since then, the FDA has acknowledged the importance of evaluating quality of life-based outcomes, stating: “improvement in neurocognitive function or delay in neurocognitive progression are acceptable endpoints” [24]. Outcomes in high-grade glioma starkly contrast to patients with meningioma, who often live many years with relatively few symptoms. Therefore, meningioma outcomes can be meaningfully described by overall survival or progression-free survival.

In addition to being clinically meaningful, an outcome measure must also be generalizable to the intended population. How specifically the study population is described in the study question often sets these parameters. For example, an outcome measure for health-related quality of life in patients with recurrent GBM could be a validated questionnaire for particular patients: cognitively intact patients with recurrent GBM after resection. For this reason, the EORTC QLQ-C30, as discussed above, is a health-related quality of life measure with excellent validity in the general cancer population yet might not be sufficient for an outcome measure in patients with GBM [25]. Similarly, in the context of meningioma, the Quality of Life in Epilepsy survey may be considered valid in patients who experience seizures as a result of meningioma, but not in patients with asymptomatic meningioma.

Limitations of the actual instrument must also be considered, such as when using self-reported measurement techniques. These include cultural and/or language barriers for certain patient populations. Appropriately describing measurement scale and score interpretations are also important for clinical context. For example, on the classic Eastern Cooperative Oncology Group Performance index, a single point difference describes the difference between a patient who is capable of self-care and “up and about more than 50% of waking hours” and a patient who is capable of only limited self-care and “confined to a bed or chair more than 50% of waking hours” [25].

**Figure 2:**
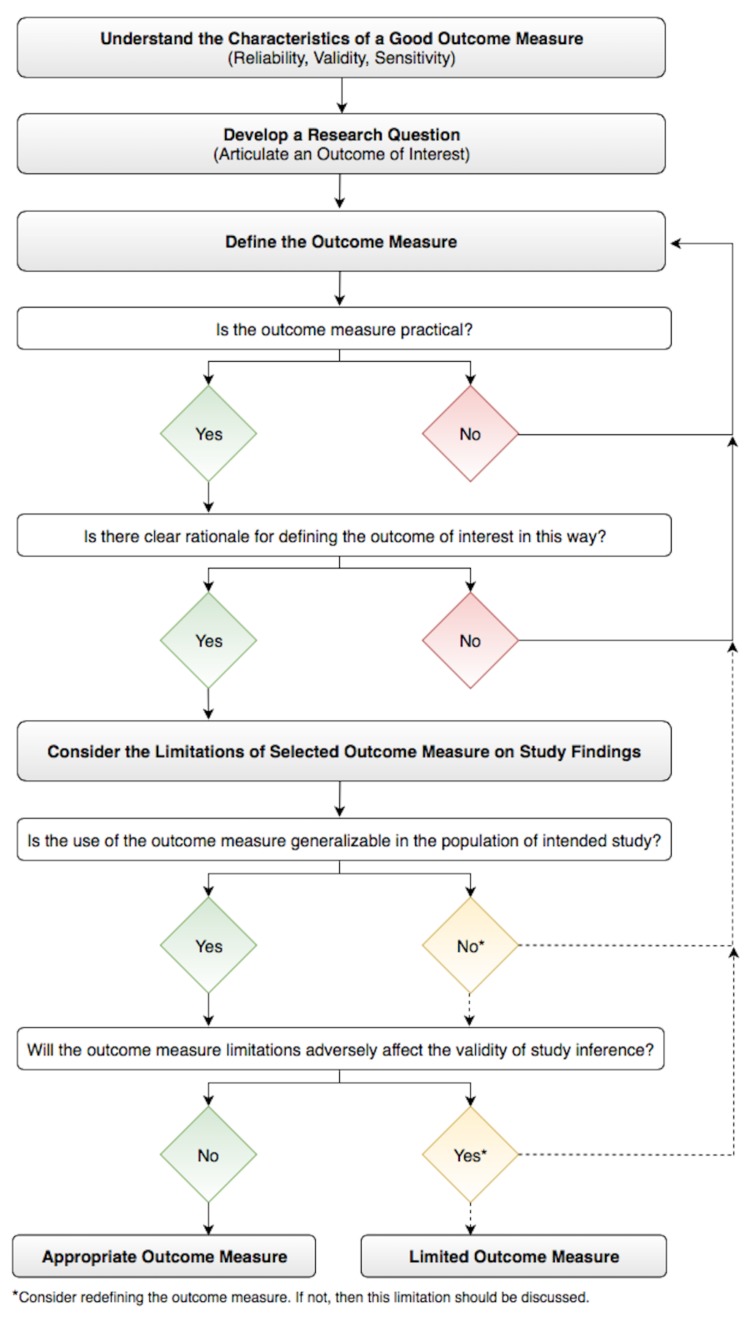
Workflow for Selecting an Appropriate Outcome Measure

Finally, it is always critically important to reflect on a study ask whether results from the outcome measure answer the research question. The limitations of the outcome measure should be identified, and whether these limitations affect the validity of the study inferences and conclusions. A useful practice is thoroughly hypothesizing how the limitations could affect the study findings (i.e., away or towards the null hypothesis) and convey these considerations accordingly. In the context of the above four-part framework, this approach enhances study design and ultimately helps identify a meaningful study outcome measure (Figure 2).

## Conclusions

It is often challenging to identify an appropriate study outcome measure, especially in a field as complex and rapidly evolving as neurosurgery. The appropriateness of the selected outcome measure can be guided by understanding the characteristics of a good outcome measure, developing a pointed research question, defining an outcome measure, and considering important limitations of said outcome measure. Following evaluation of an appropriate outcome, methodology for appropriate study design can begin, which is a topic for further discussion. By considering the fundamental elements of outcome measures, this four-part framework encourages a thoughtful approach for determining if an outcome measure is valid, practical, and ultimately meaningful.
